# Monthly Reports on Epidemics and the Sanitary Condition of Chicago

**Published:** 1854-11

**Authors:** N. S. Davis

**Affiliations:** Prof. in Rush Med. College


THE NORTH-WESTERN

MEDICAL AND SURGICAL JOURNAL.

NEW SERIES.

VOL. III. NOVEMBER, 1854. NO. 11.

### ORIGINAL COMMUNICATIONS.

ART. I.—*Monthly Reports on Epidemics and the Sanitary condition of Chicago*, prepared for the Cook County Medical Society at the Meeting in November, 1854. By N. S. DAVIS, M. D., and Prof. in Rush Med. College.

The last report, made by your committee, had reference to the diseases prevalent through the month of August. Not having been able to attend the meeting of the Society on the first Tuesday in October, no report was made for September, and hence the present paper will contain a brief review of two months.

Having in the former reports traced somewhat in detail the progress of cholera and its connection, as far as possible, with the appreciable conditions of the atmosphere, we shall at present occupy very little time or space with that subject. At the close of our last report, the cholera had ceased its ravages as an epidemic, but sporadic cases continued to occur in all sections of the city, causing from two to ten deaths per day. During the first three weeks of September, the weather was moderately cool, and for the most part the atmosphere was dry. During this time the average number of cases and deaths from this disease gradually decreased. This was so much the case, that for several days previous to the 21st, I met with no well marked case of the disease.

On Friday, September 22d, I learned of four deaths from cholera within the compass of two blocks, between Randolph and Lake streets, west of the West Side Market House. These had all been attacked during the evening of the 21st and were either dead or in a state of complete collapse before morning. On inquiring of physicians, I learned that several other cases occurred at the same time, chiefly in the West division of the city. The number of cases and deaths occurring on the 21st and 22nd, was sufficient to indicate a sudden and well marked increase in the prevalence of the disease. A few days later the same increase of cases and deaths was found to exist in the South and North Divisions; and it continued with little or no abatement until the 13th of October. The whole number of deaths during the month of September from all diseases was 560 ; of which 240 were reported to have resulted from cholera. During the first 13 days of October the number of deaths from all diseases was 190, of which 77 were attributed to cholera. During the week following the 13th the attacks became much less frequent, and for some time past I have not met with a single well-marked case. I have stated that during the first three weeks of September the weather was moderately cool and dry, and the prevalence of cholera slowly diminished. On the afternoon and evening of the 21st of the month there was a notable change. The atmosphere became very nearly saturated with moisture and the night warm. This state of the atmosphere continued two weeks. The temperature was at no time very high, neither was there at any time more than a slight sprinkling of rain, yet the proper tests showed constantly a high degree of saturation ; and some days the air was filled with a visible mist or fog. It was during this time that the cholera attacks became again so numerous as to cause considerable uneasiness in the public mind, and several well known citizens became its victims. During the third week in October the weather became cooler, accompanied by one or two copious rains, which for the first time thoroughly saturated the soil with fresh water. Since then the atmosphere has been clear, dry and pleasant, accompanied by a rapid improvement in the public health. The increase of sickness which was noticed during the last part of September and the first of October, was undoubtedly caused in part by the increased influx of emigrants from Europe and the rapid return of many from other parts of the Union under the impression that the cholera had entirely ceased in the city. It is worthy of note also that the specific character and phenomena of most of the attacks which have occurred during the last period of the prevalence of the cholera, was somewhat different from those that were noticed during the summer. A smaller proportion of them were *rapidly* fatal, and there was manifested a much greater tendency to stop vomiting and purging before complete collapse, and to result in a very low grade of secondary typhoid fever, which it was often extremely difficult to remove. There were many cases also, that presented the copious vomiting, purging, cramps, and rapid prostration of ordinary epidemic cholera; but the matters vomited, instead of presenting the appearance of rice-water, were, throughout, thin as water and of a deep *green* or *yellow* color.

Although such modifications were seen in many of the cases, yet there were some as rapidly fatal as at any previous part of the season. I recollect well the case of an Irishman who had been in the city only a few days. He rose in the morning, eat his breakfast, and went to his work in a marble yard near by.—He shortly after felt sick, returned to his boarding house, and in less than one hour, he was in a state of complete collapse, and before noon life was extinct. I learned, however, that he had had serous diarrhœa during the last part of the night and early morning, but without mentioning it to any one. In one particular the following case may be worthy of recital:

Miss C., a school girl, aged 13 years, large size and fleshy, was attacked with active cholera on the evening of Oct. 10th. I was called to visit her in the night. Found her vomiting and purging frequently, the matter discharged being entirely serous or rice-water Her eyes were sunken and the skin underneath of a leaden or purple color; the skin on the extremities shriveled and cold puse very small and frequent; the voice husky; extreme thirst for cold water; frequent cramps in the extremities; and great restlessness. I directed strong sinapisms of mustard to be applied over the epigastric region, the spine and the extremities; and a powder consisting of calomel 2 grs. acetate of lead I gr., and acetate of morphine ¼ gr., to be given *immediately after every* paroxysm of vomiting, and if the vomiting ceased, to be continued every hour until the diarrhœa ceased also. Her drinks were restricted to coffee and cold water given alternately in table-spoonful doses, and small pieces of ice to hold in the mouth. The next morning I found her still with a cold and corrugated skin, sunken eyes, husky voice, rapid and feeble pulse; but the vomiting, purging and cramps had ceased. As she was wakeful and restless, I continued to give her morphine in doses of ¼ of a gr. combined with one grain of calomel, every three hours, and spts. nit. dulc., half a tea spoonful between the powders. Up to this time there had been no secretion of urine, and the thirst remained unabated At evening she had a moderate evacuation of urine, and two small discharges from the bowels consisting of a serous fluid of the color of strong coffee with black flakes floating in it. There was also more reaction, the skin being warmer, the cheeks slightly flushed, but the pulse, thirst, &c., the same as in the morning. I now directed her the following, viz.:

℞ Sulp. quinine 12 grs.

Pulv. G. camphor, 5 grs.

Mix and divide into five powders, one to be taken every two hours. Beef-tea had been given in small quantities frequently throughout the day, together with some coffee. These were continued. I was in hopes the quinine and camphor would give more force and steadiness to the heart's action, and thereby render the pulse fuller and slower. In this, however, I was disappointed. The next morning the pulse still remained small and quick, at least 150 per minute, the skin around the eyes and on the extremities of a purplish color, but warm; the cheeks deeply flushed and hot; considerable thirst; the tongue coated and dry; slight wandering of mind at times; and great restlessness. She had passed a small amount of urine and one or two discharges from the bowels very similar to that of the day previous. Believing that no healthy capilary circulation or functional action could be established while the pulse continued so rapid and feeble, and the patient presenting the appearance of sinking soon into a most dangerous typhoid condition, I determined to try the effects of a direct and powerful sedative. All the previous remedies were discontinued except the animal broth, and the following, viz.:

℞ Sat. tinct. veratrum viride, ℥i.
Tinct. opii et camph. ℥i.
Spts. nit. dulc. ℥i.

Mixed, was given in doses of a teaspoonful (one fluid drachm) every four hours, and the face add neck sponged over frequently with cold water.

At the end of twenty-four hours, I found the pulse reduced below 90 per minute, and more full; the cheeks were less flushe, and the color of the skin improved. The patient had slept more quietly, during which a slight perspiration appeared on the forehead. She had vomited a little green fluid once or twice, and had discharged from the bowels once, the evacuation being less thin, and of a greener color, instead of black.

I continued the same treatment, only diminishing the dose of the medicine to half a teaspoonful, given at the same intervals of time. During the succeeding twenty-four hours there was no vomiting or intestinal discharges; the pulse became natural in frequency; the thirst ceased; the urinary secretion was free and natural; and the countenance good, with some appetite. From this time the patient took very little medicine of any kind, and rapidly recovered. The point of interest in this case was the extreme rapidity of the pulse, continuing after the evacuations had ceased, and the apparently prompt influence of the veratrum in controling it, and arresting the development of a severe secondary typhoid fever. Those who have had much to do with the treatment of cholera have not unfrequently met with cases in which the discharges cease before entire collapse or loss of pulse at the wrist, but in which the pulse becomes extremely rapid, small, and feeble, accompanied by just enough re-action to give warmth to the surface over the face and trunk, with remaining coldness and lividity of the extremities, and nearly suppressed secretion of urine.

So far as my observations go, such patients linger from one to six days in a typhoid cond tion, with occasional vomiting of a green liquid, and then die; though a few slowly recover. Regarding the rapidity of the pulse in such cases as depending on extreme exhaustion, I have generally endeavored to support the patients by moderate stimulants and nutritious drinks. But the large proportion of deaths with those who get into the state here described, determined me to use the veratrum viride in the foregoing case, for the purpose of directly diminishing the frequency of the heart's action. The result was such, that I shall try it again, if I chance to meet with other cases presenting the same condition of the circulation. In endeavoring to improve our stock of knowledge in regard to the successful treatment of cholera, we shall, doubtless, gain more by studying carefully the varying indication which spring up in the progress of different cases, and the means best calculated to meet those indications, than by the vain search after new remedies which may be applicable to all cases and stages of the disease.

*Dysentery.*—During the two months embraced in this report, the prevalence of Dysentery in this city has very much exceeded, in the number of attacks, that of Cholera. It has prevailed, more or less, among all classes and ages, but the emigrants, those who have recently commenced their residence here, and the children, have suffered most. As a generel rule, the disease has been mild and easily controlled by proper remedies; but there have not been wanting cases of the most obstinate duration, and others of the most malignant or rapidly fatal character. The great majority of cases that I have met with, have commenced with rigors, followed by fever, pain in the head, loins, and extremities, together with frequent discharges of mucous, more or less mixed with blood, and accompanied by severe griping pains and tenesmus; agreeing in all respects so fully with the descriptions of the disease given by well known authors, that any details given here would be a waste of valuable time. A few cases have been observed in which the disease manifested distinct periodicity, and readily yielded to the use of Quinine and Opium in anti-periodic doses.

The most dangerous class of cases have been characterized at the beginning, by pretty *copious* and frequent discharges of a serous fluid, mixed with sufficient dark colored blood to give the whole a dark red appearance. The patients complain of comparatively little pain or tenesmus, but feel a great sense of oppression and distress in the epigastrium, and extreme debility. The general febrile action is feeble, the extremities often being cool; the pulse soft and not more than 90 per minute; the countenance of a leaden or dingy hue and the expression dull; the mouth dry; the voice husky; the respiration feeble, and the mental operations slow. If the disease continues uncontrolled from three to five days, the tongue becomes more dry and covered, especially in the middle and back part, with a brown coat; the upper lip becomes retracted and the teeth covered with sordes; the pulse more frequent and feeble; the mind more dull and disposed to wander; and the discharges continue frequent, considerable in quantity, and consisting of a large proportion of very dark colored blood, and emitting an extremely offensive odor. Generally there are flakes and shreds of Lymph mixed with the discharges. Some of the cases terminate in fatal exhaustion between the end of the first and the middle of the second week. Others terminate still earlier by the supervention of true cholera, or rice-water, discharges with vomiting, and almost immediate collapse. I have seen only three or four of this class of cases. The following case will illustrate both the symptons and treatment of that form of the disease which we have been describing.

Mr. B., a native of Ireland, aged about 30 years, was admitted into the Mercy Hospital during the second week in October. He had been sick five or six days. His face was of a purplish red color; his tongue covered with a dry, brown coat, upper lip retracted, and teeth covered with dark sordes; mind very dull and inactive; pulse 90 per minute and soft; skin dry snd rough; abdomen full and tympanitic; with very frequent discharges from the bowels, consisting almost wholly of dark blood, emitting a very offensive smell, and amounting to very considerable in quantity. The discharges were frequently accompanied by much pain, and an imperfect control over the sphincter of rectum, allowing the blood to escape sometimes involuntarily. Wishing to diminish the quantity of bloody discharge, I directed for the patient a powder consisting of

| | |
|---|---|
| ℞ Pulv. Opii, | 1 gr. |
| Pulv. Alum, | 4 grs. |
| Sub. Murias Hyd., | 2 grs. |

Mix and give every two hours; and between each dose give two fluid drachms of the Emulsion of Oil of Turpentine and Tinct. Opii.

Each dose of the latter would contain about 15 drops each of the Turpentine and Laudanum. The following morning I found no other change in his symptoms than a diminution of the bloody discharges. But they continued very offensive and dark colored, and the sphincter Ani was so much relaxed as to allow the discharges to occur before the patient could be gotten up or out of bed.

The powders were discontinued, and in their place, I directed a tea-spoonful of the following, viz:

| | |
|---|---|
| ℞ Nitric Acid, | ʒi. |
| Tinct. Opii, | ʒii. |
| Strychnine, | 1 gr. |
| Water, | ℥ii, mix. |

The Emulsion was continued as before, and the patient allowed a liberal supply of animal broth, well seasoned with salt. He was continued steadily on the use of these remedies for three days, during which time the discharges ceased to contain blood, and become fecal, though still much too frequent and liquid. His countenance also improved; the tongue became more moist, and the teeth less covered with sordes; and the mind more active. As the discharges remained quite frequent, though destitute of blood, and much improved in their nature, I directed a pill consisting of nitrate of silver one third of a grain, and opium one grain, to given two hours until the bowels should become quiet. We continued the pills about three days when the evacuation nad become so much less frequent that they were continued only three times a day. At the present time thre remains still a morbid senseitiveness of the mucous membranes manifested by three or four thin fecal evacuations every 24 hours. The chief points of interest in this case were, the large quantity of very dark and grumus blood contained in the discharges; the relaxation or loss of power over the sphincter ani; and the apparent beneficial action of strychnine in removing the latter, I have had occasion several times to resort to the strychnine for the same purpose, in the advanced stage of typhoid fever and dysentery, and it has seldom failed to produce good effects. In a considerable number of cases of dysentery, accompanied by an unusual amount of blood in the discharges, together with much prostration, I have found the pulv. alum, in doses of from three to five grains, combined with opium, one of the most efficient astringents we possess. It is less sedative or debilitating than the acetas plumbi, and equally effectual in diminishing the quantity of the discharges.

*Continued Fever.*—During the last two months I have met with only four cases of that variety of continued fever called *Typhus;* and these were all in the persons of emigrants brought to the Mercy Hospital soon after their arrival in this city. There was nothing peculiar in their symptoms or treatment.

They were received into the same wards with other patients, but no other cases occurred among the inmates.

The ordinary typhoid fever has increased almost in the same ratio with the decrease of cholera and dysentery. Of thirty-two cases, concerning which I kept memoranda, twelve occurred during the month of September and the remaining twenty in October. In this list are not included those cases of typhoid disease following genuine attacks of cholera or cholera morbus. In referring to the class of individuals attacked, I find that nearly all were between the ages of 18 and 45 years; and 21 out of the 32 had been residents of the city between one and six months. Only a small proportion of the whole were emigrants recently from Europe, but more than half of the number were young men from other States, who had commenced their residence here since the first of March last. During the month of September and the first half of October, nearly all the attacks of typhoid fever commenced with general lassitude and a moderate diarrhea, lasting from two to six days, when for one day the discharges would become more frequent, accompanied by pains in the head, back, and abdomen; a pulse between 90 and 100 per minute; skin dry and moderately hot; the tongue covered with a dirty white coat, most thick along the middle; sometimes the edges red, and the whole mouth deficient in moisture. This would characterize the onset of the disease, or the period when the patient takes to his bed. In some instances the gastric and intestinal irritation at the commencement was more severe, giving rise to both diarrhœa and vomiting. These, however, lasted only one or two days, when generally the gastric irritation subsided, and the diarrhœa consisted of only three or four thin fecal discharges per day. But as the intestinal discharges became less frequent, the general febrile action increased, the pulse becoming quicker and more full, the skin more hot, the face more flushed, the mouth more dry, and the coat in the middle of the tongue, appearing more dry and brown with redder edges, the abdomen rotund and slightly tympanitic, and the mind dull, with slight indications of delirium, especially during the night. The subsequent course of the disease has not differed from ordinary intestinal typhoid fever, as described in the books. During the last half of October the gastric and intestinal symptoms were much less prominent at the commencement of the attack; and in a large proportion of cases, the patients complained, during the first day or two, of rigors and chilliness, with more decided pains in the head, back, and extremities. In some cases, though the rigors did not continue after the second day, yet the fever was distinctly remittent, presenting a well marked exacerbation every afternoon and evening. So distinct were the exacerbations that in three cases full antiperiodic doses of quinine and opium were given, but in every instance it failed to cut short the disease and establish convalescence. Though I have in no case been able to *arrest* the fever by the use of quinine, yet in several cases which manifested distinct morning remissions, I have given four grains in a tea-spoonful of the emulsion of oil of turpentine and laudanum, at 6 and 9 o'clock A. M., continuing the emulsion without the quinine during the remainder of the 24 hours, and with decided benefit. Most of the cases that have come under my care have been mild and easily conducted to a perfect convalescence Of those that came under my care during the first week of their sickness, only one died. In the hospital where many patients are received after the fever has been existing from two to three weeks, three died with the disease, presenting plain evidence of ulcerations in the mucous membrane of the ilium. The one case that terminated fatally after having been subjected to treatment early, was that of a young man who was attacked first with diarrhœa, for which he took some very heating medicine from an eclectic physician.

The medicine checked the diarrhœa very quickly, but it was followed immediately by an increase of fever and pain in the head and abdomen. I found him the following day with all the symptoms of a severe continued fever with intestinal irritation. At the end of a week the fever subsided, the tongue became clean, the fecal evacuations very nearly natural, and all the usual symptoms of entire convalescence. But the patient, instead of gaining strength, continued dull and weak, and a slight tenderness remained over the right iliac region. He continued in this state of apparent convalescence eight or ten days, very slowly improving in appetite and strength, when he was seized with sudden and severe pain in the abdomen, followed by rapid distension and tenderness, with a feeble and very quick pulse, and death ensued in 48 hours. Though no post mortem examination was made, yet there could be very little doubt but perforation of the intestine and consequent peritonitis was the cause of the fatal result. The members of the society are aware that the use of the veratrum viride in the treatment of typhoid fever, has attracted considerable attention in certain places during the last two years. I have made it the subject of special attention during the present season, and have found a certain class of cases in which its medicinal effects are very valuable. These cases are such as present, early after the attack a rapid pulse and hot skin, indicating an unusual grade of irritative excitement in the circulation. The following case will illustrate the class to which I refer, and the mode of prescribing the remedy:

Mr. B., a native of Ireland, aged 35 years, had been in the country, and was there attacked with fever, preceded with lassitude and rigors. He was induced to take freely of Cayenne pepper, brandy and quinine. Continuing sick, however, he was, at the end of the first week, sent into the city, and received into the Mercy Hospital. I found him with a deep red flush over the whole face; the eyes suffused; the lips, mouth, and tongue dry, the latter being brown along the middle and red at the edges; the mind wandering; the skin hot; the pulse 120 per minute and moderately firm; the respiration hurried; the bowels quiet; and the urine scanty.

I directed for the patient a powder containing Sub. Murias Hydrag. two grains, Pulv. Doveri and Bi Carb. Soda, each five grains, to be given every four hours, with a tea-spoonful of Spts. Nit. Dulc. between. The surface, especially of the face and upper part of the trunk, were to be sponged over with cold water. This treatment was continued forty-eight hours without any decided alteration of the symptoms. The tongue became more dry and brown, the skin remained hot, and the pulse more than 120 per minute. Owing to the continued rapidity of the circulation, and active grade of febrile excitement, I discontinued the powders and Spts. Nit. Dulc., and gave the following, viz:

| | | |
|---|---|---|
| ℞ Tinct. Veratrum Viride | - - | ʒi |
| Tinct. Opii et Camph. | - - | ℥j |
| Spts. Nit. Dulc. | - - - | ℥j mix |

and give one teaspoonful every two hours; and allow a small quantity of beef-tea for nourishment. The following morning the pulse was reduced to 80 per minute, the skin was cool and moist, the mind less delirious but inactive, but the surface of the tongue remained dry, and the bowels had moved three times, the discharges being thin and brown. The Veratrum mixture was continued at intervals of once in 6 hours, and a teaspoonful of the emulsion of turpentine and laudanum given between. This was continued for three days, during which the pulse remained below 80 per minute, and the general febrile action seemed entirely overcome. The tongue also became more moist and of a better color, and the discharges from the bowels more healthy. The veratrum was now omitted and the emulsion alone continued two or three days longer, when complete convalescence was established. The rapid subsidence of the fever and the reduction of the pulse exhibited in this case, I have seen follow the use of the Veratrum Viride in almost every instance, in which the fever in its early stage was accompanied by unusual frequency of pulse, without excessive intestinal irritation.

But its beneficial effects have been limited to these cases alone. In those cases manifesting but a moderate frequency of pulse, a depressed state of the organic actions, and general dulness or diminished sensibility, I have derived no benefit from its use. And in all cases of typhoid fever it is necessary to guard against its tendency to disturb the stomach and bowels. To effect the latter purpose, I generally exhibit it in combination with camphorated tincture of opium.

I will mention but one other disease in this report.

*Puerperal Fever.*—Between the 15th and 25th of September, I met with four cases of well marked puerperal peritonitis.

These cases occurred so near together that I at first feared that it was commencing an *epidemic* prevalence. But on inquiring of other practitioners, I found no corresponding prevalence of it in their practice, and no other cases have occurred since in my own. I was, during the period named, attending one or two cases of severe erysipelas, and it is possible that a poison might have been communicated to the females, or the occurring of the four cases within a few days of each other, might have been purely accidental. The first case occurred in a female of delicate constitution, and who had manifested strong indications of the "*nursing sore mouth,*" during the whole period of gestation. It was her first child, and the labor was rather protracted. She appeared to be doing well, however, until the fourth day after delivery, when she was seized with chills, followed by a hot skin, very rapid pulse, hurried breathing, anxiety, restlessness, and severe pain in the lower part of the abdomen, with acute tenderness to pressure, and a rapidly increasing swelling of the abdomen. At the same time the lochia and the milk were suppressed. She was directed a powder, consisting of Opii 2 grs., and Sub. Murias Hydrarg., 4 grs., every two hours, with anodyne fomentations to the abdomen. At the end of 48 hours the progress of the disease was arrested and the symptoms favorable. Wishing to avoid as far as possible the effects of mercurials, on account of the constitutional delicacy of the patient, and having heard the muriated tinct. of iron spoken of in very high terms as a remedy in this disease, I directed it to be given instead of the powders, in doses of 20 drops, diluted with sweetened water. Its exhibition, however, was quickly followed by gastric irritation, with increased pain in the lower part of the abdomen and great restlessness. She was put upon the use of sedative doses of opium alternated with the tinct. of Digitalis and spts. nit. dulc., and ultimately recovered. The second case occurred after an ordinary labor, but was quickly overcome by a few full doses of opium combined with calomel. The third case occurred the third day after a premature labor, occurring about the fifth month of pregnancy. She was treated with opium and calomel and fomentations, as in the two previous cases, and convalescence was pretty well established about the fifth day after the attack. She continued apparently recovering two or three days, when she was suddenly attacked with cholera, sunk rapidly into collapse, and died in a few hours. The fourth case was that of a lady who had been several times delivered at the full period of gestation, but always with still-born children. This latter result was owing to the fact that her children invariably presented unnaturally. In this, her last confinement, the posterior part of the left *scapula* presented, and was the only part that could be reached with the finger. She was delivered without much difficulty or delay by introducing the hand and bringing down the feet.

She continued to progress very favorably until the commencement of the third day, when she was seized with chills, followed by a hot skin, deeply flushed face, a pulse up to 150 per minute, breathing hurried, countenance anxious, abdomen full, tympanitic and very tender, with constant severe pain in the lower part of the back and abdomen. The lochial discharge was also suppressed. I gave her every two hours a powder, composed of Pulv. Opii 2 grs and calomel 3 grs., with warm hops over the abdomen. After the first 24 hours, the stomach began to reject every form of opiate that was given. Still all the bad symptoms continued; the pulse was very quick, the abdomen very full and tender, and the patient extremely restless. Unable to use opiates, and fearing to bleed, I resolved to try the sedative influence of the veratrum viride. I accordingly directed the following, viz:

℞ Tinct. Opii et Camph. - - ℥ij

Tinct. Veratrum Viride - - - ʒi mix, and give one fluid drachm every two hours. This was retained by the stomach, and in 12 hours the patient was more quiet, and the pulse less frequent. The medicine was continued at intervals of three hours, and in 12 hours more, the pulse was below 90 per minute, the face pale, the skin cool and covered with moisture, the abdomen less tender, and the patient tranquil. Fearing too much sedative action, I omitted the veratrum, and directed moderate doses of quinine and camphor. In less than 24 hours, however, the fever, quick pulse, restlessness, and abdominal tenderness began again to increase. She was immediately directed to re-commence the use of the tinct. of veratrum viride and paregoric in the same doses as before, and at intervals of three hours Its effects were as promptly beneficial as before, and by continuing it in smaller doses and at longer intervals for two or three days, complete convalescence was established, and the patient made a rapid recovery This is the first and only case of child-bed fever in which I have used the veratrum viride. Whether it will prove a valuable agent for combatting this much dreaded disease, further trials must determine.

